# Clinical and Therapeutic Implications of Epstein–Barr Virus in HIV-Related Lymphomas

**DOI:** 10.3390/cancers13215534

**Published:** 2021-11-04

**Authors:** Miriam Verdu-Bou, Gustavo Tapia, Agueda Hernandez-Rodriguez, Jose-Tomas Navarro

**Affiliations:** 1 Lymphoid Neoplasms Group, Josep Carreras Leukaemia Research Institute, Can Ruti Campus, 08916 Badalona, Spain; mverdu@carrerasresearch.org; 2 Department of Pathology, Germans Trias i Pujol Hospital, Universitat Autònoma de Barcelona, 08916 Badalona, Spain; gtapia.germanstrias@gencat.cat; 3 Department of Microbiology, Germans Trias i Pujol Hospital, Universitat Autònoma de Barcelona, 08916 Badalona, Spain; ahernandez.igtp.germanstrias@gencat.cat; 4 Department of Hematology, Institut Català d’Oncologia-Germans Trias i Pujol Hospital, 08916 Badalona, Spain; 5 Department of Medicine, Universitat Autònoma de Barcelona, 08916 Badalona, Spain

**Keywords:** Epstein–Barr virus, human immunodeficiency virus, HIV-related lymphomas

## Abstract

**Simple Summary:**

Epstein–Barr virus (EBV) is involved in lymphomagenesis, especially lymphomas affecting populations with immunodeficiencies, such as people living with HIV (PLWH). The pathogenic roles of EBV in lymphomas arising in PLWH are mediated by several viral proteins, as well as cooperation between EBV and HIV. The presence of EBV in these lymphomas conditionate some of their epidemiological, pathological, and clinical characteristics, as well as their prognosis. In this article, the authors review the different EBV-associated lymphomas affecting PLWH, analyzing the influence of EBV on the epidemiology, etiopathogenesis, clinical features, treatment, diagnosis and prognosis of each lymphoma subtype. Furthermore, new EBV-targeted therapies currently under development for some lymphomas are discussed.

**Abstract:**

The incidence of lymphomas is increased in people living with HIV (PLWH). Aggressive B-cell non-Hodgkin lymphomas (NHLs) are the most common and are considered an AIDS-defining cancer (ADC). Although Hodgkin lymphoma (HL) is not considered an ADC, its incidence is also increased in PLWH. Among all HIV-related lymphomas (HRL), the prevalence of Epstein–Barr virus (EBV) is high. It has been shown that EBV is involved in different lymphomagenic mechanisms mediated by some of its proteins, contributing to the development of different lymphoma subtypes. Additionally, cooperation between both HIV and EBV can lead to the proliferation of aberrant B-cells, thereby being an additional lymphomagenic mechanism in EBV-associated HRL. Despite the close relationship between EBV and HRL, the impact of EBV on clinical aspects has not been extensively studied. These lymphomas are treated with the same therapeutic regimens as the general population in combination with cART. Nevertheless, new therapeutic strategies targeting EBV are promising for these lymphomas. In this article, the different types of HRL are extensively reviewed, focusing on the influence of EBV on the epidemiology, pathogenesis, clinical presentation, and pathological characteristics of each lymphoma subtype. Moreover, novel therapies targeting EBV and future strategies to treat HRL harboring EBV are discussed.

## 1. Introduction

People living with HIV (PLWH) are at higher risk of developing aggressive B-cell lymphomas than the general population, and B-cell non-Hodgkin lymphoma (NHL) is currently the most frequent AIDS-defining cancer (ADC) [1,2]. Among NHL, diffuse large B-cell lymphoma (DLBCL) is the most common, including primary central nervous system lymphoma (PCNSL), followed by Burkitt lymphoma (BL). The frequency of other lymphoma subtypes, such as plasmablastic lymphoma (PBL) and primary effusion lymphoma (PEL) is lower, but they more frequently affect PLWH [3]. While the incidence of aggressive B-cell NHL has decreased in PLWH since the widespread use of combined antiretroviral therapy (cART), lymphoma is still an important cause of morbidity and mortality among this population [4,5]. On the other hand, although Hodgkin lymphoma (HL) is not considered an ADC, its incidence increased among PLWH during the first years after the introduction of cART [3,6], and eventually has remained stable or with only a slight increase [7,8].

The pathogenesis of HIV-related lymphomas is complex and influenced by immunosuppression and coinfection with oncogenic viruses, mainly human herpes virus 8 (HHV-8) and Epstein–Barr virus (EBV), two human γ-herpesviruses that establish latency in the host B-cell nucleus. However, the prevalence of EBV is higher in HIV-related lymphomas (HRLs) and is therefore implicated in the pathogenesis of these lymphomas [9,10,11,12]. Some components of the virus related to the latent state, such as EBV latent membrane proteins (LMP1s and LMP2A/B), EBV-nuclear antigens (EBNA1, EBNA2, EBNA3A, EBNA3B, EBNA3C, EBNA leader protein (EBNA-LP), EBV-encoded small RNAs (EBERs), and microRNAs (miRNAs) play important roles in lymphomagenesis [13]. The EBV latency type differs among HRLs, suggesting that EBV may regulate different processes related to B-cell transformation and evasion of the immune system [14,15,16,17,18,19]. The EBV-encoded miRNAs have a role in the lymphomagenesis, regulating different cellular pathways such as apoptosis, proliferation, immune recognition, and microenvironment [20]. EBV-miRNAs are located in two regions of the viral genome: the BamHI-A region rightward transcript (BART) and BamHI-H rightward fragment 1 (BHRF1) [21]. The expression of viral miRNAs differs according to the latency type; while BART miRNAs are expressed in all latency types (mainly in latency I and II), BHRF1–3 miRNAs are expressed almost exclusively in the latency III type [21]. During the latent state, EBV can intermittently reactivate, expressing lytic genes that are also involved in lymphoma development [22].

Furthermore, cooperation between HIV and EBV in HRLs has been speculated, with HIV likely contributing to the generation of a permissive microenvironment for EBV infection, and the differentiation and survival of infected B-cells [18,23,24].

In addition to the presence of EBV in lymphoma tissue, the virus can be detected in peripheral blood of patients with lymphoma. Some studies have suggested the usefulness of EBV load as a lymphoma biomarker with diagnostic and prognostic implications in PLWH [19,25,26,27].

There is great interest and a need to develop new EBV-targeted therapies for the treatment of patients with EBV-driven lymphomas. Multiple EBV-target strategies have been studied in HRLs in preclinical studies, but few clinical trials have been carried out [28].

This article is a review of the state of the art of the implications of EBV in different clinical, epidemiological, and etiopathogenical aspects of HRL, as well as a description of the EBV-targeted therapies currently under development for the treatment of these lymphomas.

## 2. Implications of Epstein–Barr Virus in the Different HIV-Related Lymphoma Types

### 2.1. Diffuse Large B-Cell Lymphoma

**Figure 1:**
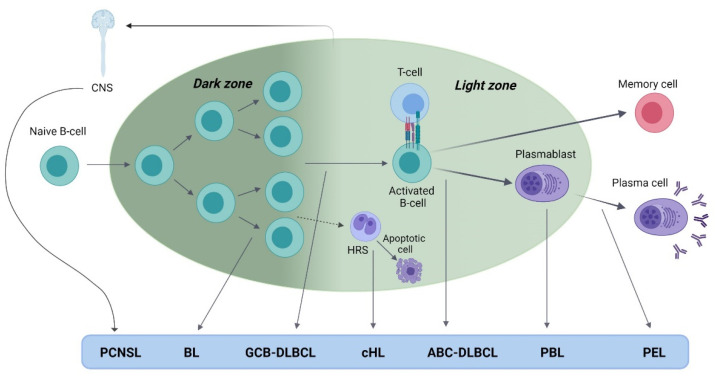
EBV-associated HIV-related lymphoma development during B-cell differentiation in the germinal center. Burkitt lymphoma (BL) and germinal center B-cell diffuse large B-cell lymphoma (GCB-DLBCL) have a germinal center (GC) origin. Primary central nervous system lymphoma (PCNSL) has a late B-cell origin with both ABC and GC B-cell features. Hodgkin lymphoma (HL) is originated in a preapoptotic GC B-cell. ABC-DLBCL develops after the activation of the B-cell through the interaction with antigen-presenting cells. After this event, cells can differentiate into memory B-cells or plasma cells. During plasma cell differentiation, activated B-cells are first differentiated to plasmablast, which is the cell of origin of plasmablastic lymphoma (PBL). Primary effusion lymphoma (PEL) is originated in post-GC B-cells with plasmablastic differentiation.

Diffuse large B-cell lymphoma is an aggressive B-cell neoplasm that can be classified, according to the cell-of-origin (COO), into germinal center B-cell (GCB) and activated B-cell (ABC) [29]. Two entities have classically been considered, systemic DLBCL and DLBCL with exclusive involvement of the central nervous system (Figure 1) [30]. Although the WHO classification reserves the term primary DLBCL of the central nervous system (PCNSL) for immunocompetent patients, PLWH typically have DLBCL with exclusive involvement of the CNS, which is considered an AIDS-defining condition, thus the term PCNSL is commonly found in the literature in the HIV setting [31].

#### 2.1.1. Epidemiology

Systemic DLBCL is the most common NHL in both PLWH and the general population. Although its incidence in PLWH has diminished since the widespread use of cART, DLBCL is still an important cause of morbidity and mortality among this population [15,32,33,34]. EBV infection is present in 30–50% of HIV-related DLBCL cases, a frequency higher than that of the general population [15,19,35,36,37,38]. According to the COO, EBV is more frequently found in ABC-DLBCL (44–74%) than in GCB-DLBCL (13–25%) [15,37,39]. To our knowledge, in PLWH with DLBCL, no differences regarding age and gender have been reported between EBV-positive and EBV-negative cases [19,37,39,40].

PCNSL is an infrequent lymphoma affecting 15 per 100,000 persons-year in PLWH [32]. HIV is considered a risk factor in this lymphoma, but its incidence has dramatically decreased in the cART era [31,32,41,42]. Nearly all cases (80–100%) of HIV-related PCNSL are associated with EBV [27,43,44,45,46].

#### 2.1.2. Etiopathogenesis

As reported by Arvey et al., the frequency of EBV latency types differs in PLWH depending on the DLBCL subtype [15]. They described that 76% of GCB-DLBCLs are associated with latency type I (LMP1−, EBNA2−), 12% with latency type II (LMP1+, EBNA2−), and 12% with latency III (LMP1+, EBNA2+) (Table 1). On the other hand, in ABC-DLBCLs, either latency types II or III were found in 30% of cases each, and latency type I in 37% of cases. These data suggest that EBV could be involved in different pathogenic mechanisms depending on the DLBCL COO subtype.

HIV-related DLBCL associated with EBV also presents high expression of Blimp1, a transcriptional repressor of *TP53*, which confers the capacity to escape from apoptosis and deregulate B-cell differentiation [19]. Furthermore, EBV-positive HIV-related DLBCL frequently expresses CD30, which is an NF-кB promoter [19]. It has been demonstrated that LMP1 activates the NF-кB signaling pathway and induces chronic B-cell activation, both being typical deregulated pathways in the ABC subtype [19,37,47]. Thus, EBV could have a synergistic effect on the lymphomagenesis of the HIV-related ABC subtype.

The expression of miR-BHRF1–3 has been observed in an increased frequency in primary cell lines and frozen samples from HIV-related DLBCL [48]. This miRNA targets C-X-C motif chemokine ligand 11 (*CXCL-11*), providing the cells the ability to escape from the immune system. The expression of different miR-BHRF1s have been also confirmed in frozen samples of HIV-related DLBCL [49]. In addition, these patients showed an elevated expression of miR-BARTs 15, 10-3p, 11-3p, and 14-3p compared with other EBV-related lymphomas in immunocompetent individuals [49].

Additionally, miR-BHRF1–2 inhibits *PRDM1*, preventing apoptosis and cell cycle arrest in lymphoblastoid cell lines (LCL) [50]. This EBV-miRNA could be a synergistic mechanism of the downregulation of *PRDM1* in GCB-DLBCL. Furthermore, some EBV-miRNAs such as miR-BART3, miR-BART9, and miR-BART17-5p can downregulate *BCL6*, an NF-кB repressor [51].

The expression of EBV-lytic genes has been also described in lymphomas, and emerging studies of its impact on the lymphomagenesis are in progress [22]. Cohen et al. have reported an expression of the lytic proteins BZLF1, BHRF1, and BLLF1 in immunocompetent EBV-related DLBCL that could deregulate cellular pathways and contribute to the lymphomagenesis [52].

Additionally, HIV could be involved in the pathogenesis of DLBCL in PLWH. In this regard, Liapis et al. reported increased infiltration of CD8+ cytotoxic T-lymphocytes (CTL) in DLBCL tumors with expression of LMP1 and the HIV-1 p24 protein, which is related to active HIV replication [53]. Furthermore, it has been postulated that the HIV-1-matrix protein p17 persists after antiretroviral drug suppression and acts as a cytokine for T-cell activation and promotes angiogenesis [54,55]. In addition, p17 also can increase the expression of LMP1 in primary EBV-infected B-cell lymphocytes, as described by Martorelli et al. [16]. Both p17 and LMP1 are involved in the cell growth mediated by the Akt/ERK and STAT signaling pathways, suggesting cooperation between HIV and EBV in the proliferation of malignant B-cells and the lymphomagenesis of DLBCL [56].

In HIV-related PCNSL, EBV infection affects 80–100% of cases [57,58]. In these lymphomas, EBV latency III (including LMPs, EBNAs, and EBERs expression) is the most frequent latency type [59]. This lymphoma usually affects PLWH at advanced stages of immunosuppression, with very low CD4+ lymphocyte counts (median < 50 cells/µL) and with loss of immune response mediated by CTLs [58,60]. Although EBV does not replicate in the tissue of the central nervous system (CNS), the lax immune response produced by HIV facilitates EBV infection and the migration of infected B-cells to the CNS [58,61].

#### 2.1.3. Impact of EBV on Clinical Features and Prognosis

Systemic HIV-related DLBCL usually has extranodal involvement and an advanced stage at diagnosis (III or IV) [19]. These patients usually present median CD4+ lymphocyte counts of 100–223 cells/µL [40,53,62]. The impact of EBV on the clinical features and prognosis of DLBCL in PLWH have been scarcely studied. Chao et al. found no differences in most of the clinical variables according to EBV status, but they found a significant reduction in CD4+ lymphocyte counts in EBV-positive compared with EBV-negative cases (mean 128 cells/µL vs. 248 cells/µL). These authors found that the period of time between HIV infection and the development of DLBCL tended to be shorter in EBV-positive compared to EBV-negative cases [19]. In addition, there was a trend toward a higher frequency of ABC cases in EBV-positive cases [19,63].

The impact of EBV on prognosis in HRL is a matter of controversy. In a study by Chao et al., the presence of EBV in tumoral tissue was associated with a worse overall survival (OS) [19]. However, Chadburn et al. described that the presence of EBV in lymphoma cells was not associated with poorer survival or low CD4+ lymphocyte counts [63].

PCNSL usually occurs in patients with advanced immunosuppressed status and low CD4+ lymphocyte counts (<50 cells/µL), and frequently with an AIDS-defining illness before lymphoma diagnosis [31,45,64,65,66]. In this regard, low CD4+ lymphocyte counts and high HIV load are associated with a worse outcome in patients with PCNSL [31,42]. Given that EBV coinfection is detected in nearly all HIV-PCNSL patients, the detection of EBV-DNA in cerebrospinal fluid (CSF) is a quick diagnostic tool for HIV-related PCNSL diagnosis, having a high sensitivity and specificity [45,67]. Thus, the elevated frequency of EBV infection and the advanced immunodeficiency conditions caused by HIV suggest that EBV plays a relevant role in the lymphomagenesis of PCNSL.

#### 2.1.4. Treatment

Patients with systemic HIV-related DLBCL are treated with the same regimens as the general population. The gold standard treatment used in DLBCL is rituximab, cyclophosphamide, doxorubicin, vincristine, and prednisone (R-CHOP). Additionally, it is highly recommended to add cART concomitantly with the immunochemotherapy because this strategy has been demonstrated to improve the outcome of these patients [68,69]. The recommended treatment for relapsed/resistant patients is the same as that used in the general population, and therefore they should receive second-line regimens containing drugs not included in the frontline treatment, followed by autologous stem cell transplantation (ASCT) (Table 2) [70].

There is no gold standard treatment for patients with HIV-related PCNSL. Patients with a good general condition currently receive induction treatment with high-dose methotrexate-based polychemotherapy in combination with cART [71,72]. In some patients, ASCT can be considered. Radiotherapy may be considered in patients without response to chemotherapy treatments [73].

### 2.2. Burkitt Lymphoma

Burkitt lymphoma is an aggressive NHL with a germinal center (GC) origin that can be classified as follows depending on the epidemiological characteristics: endemic BL, sporadic BL, and immunodeficiency-associated BL, which is mainly associated with HIV infection (Figure 1) [29,74].

#### 2.2.1. Epidemiology

Similar to DLBCL, PLWH have a higher incidence of BL than the general population, and BL comprises 20–30% of HRL [29,75]. HIV-related BL is more frequent among men (86.7%) with a median age of 39 years and is associated with EBV in 30–60% of cases [76,77,78,79,80]. This entity mainly develops in individuals at an early stage of HIV infection with a moderate reduction of CD4+ lymphocyte counts (200–270 cells/µL) [77,81]. Unlike DLBCL, the introduction of cART did not lead to a decrease in the number of BL cases diagnosed in PLWH, and the incidence has remained stable in recent years [82,83]. Regarding pathologic features, plasmacytoid differentiation is characteristic of HIV-related BL, and is found in 50–70% of EBV-infected cases [57,77,84].

#### 2.2.2. Etiopathogenesis

The causes and mechanisms that lead to the development of BL have not been completely elucidated. The translocation of *MYC* with the immunoglobulin heavy-chain loci (*IgH*) and inactivation of p53 are hallmarks of the pathogenesis of BL [80,85]. In HIV-related BL, *MYC* translocations and *TP53* mutations are detected in 90–100% and 60% of cases, respectively [78,80]. The translocation of *MYC* leads to constitutive activation of *MYC*, increasing proliferation, so these tumors are characterized by their high proliferative rates [57].

Among cases with EBV coinfection, latency type I (EBERs+ and EBNA1+ expression) is the most common EBV latency type (90%), although it is possible to find latency type II (EBERs+, EBNA1+, LMP1+) in a few cases (10%) (Table 1) [15,59]. LMP1 and EBNA2 have antagonistic effects to Myc in the phenotype of BL B-cells. This could be the cause of the reduced frequency of latency type II and the absence of latency type III in this lymphoma [86].

**Figure 2:**
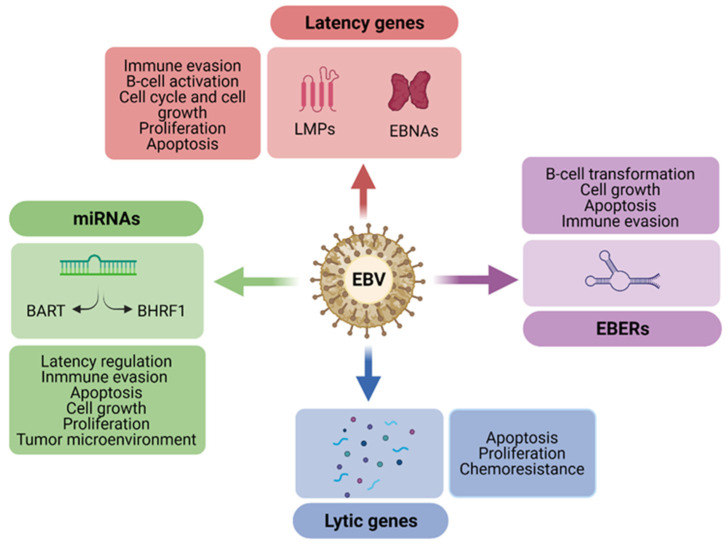
EBV genes and noncoding RNAs are involved in diverse lymphomagenic processes. EBV can prevent apoptosis and promote cell growth and proliferation via latency and lytic genes, miRNAs, and EBERs. These EBV components can also protect infected B-cells from the immune cells’ recognition via latency genes, miRNAs, and EBERs. Moreover, miRNAs can also alter the microenvironment, inducing the transformation of tumor-associated macrophages. Latency genes and EBERs are also involved in activation and transformation of B-cells. EBV may also induce chemoresistance mediated by lytic genes.

The EBNA1 protein not only maintains the EBV episomal genome in the host cells and regulates extrachromosomal replication, but may also deregulate host gene expression [17,87]. This protein has an antiapoptotic effect, since it decreases p53 stability and thus favors the survival of tumoral cells [88,89]. Furthermore, Zhang et al. described a hypermethylation of *PRDM1* (the gene that encodes the Blimp1 protein) exclusively in EBV-positive BL, inactivating this gene [90]. The overexpression of this gene results in cell cycle arrest, thus the inactivation of *PRDM1* could be beneficial for cell growth and contribute to lymphomagenesis of BL in EBV-infected cases (Figure 2) [90].

Additionally, *MYC* could alter immune system response by reducing the activity of the NF-кB pathway. This effect, in combination with the poor antigenic property of EBNA1, leads EBV-infected B-cells to acquire the ability to escape from immune system recognition [75]. Moreover, the absence of the immunogenic proteins EBNA2 and LMP1 in EBV-positive HIV-related BL could be a self-defense mechanism of the virus to remain in host cells and escape the immune system [11,16].

The EBV lytic protein, BHRF1, confers protection from apoptosis in EBV-positive BL cell lines, since it is a homolog of the antiapoptotic protein BCL-2 and negatively regulates the proapoptotic protein Bim [91,92]. This phenomenon can avoid the cell death associated with genetic alterations in *MYC*, being a cooperative mechanism of Myc-driven lymphomagenesis and favoring the chemoresistance [92]. Another lytic protein, BZLF1, directly inhibits p53 in BL cell lines and could be an alternative mechanism for the inhibition of this protein, in addition to the inactivating mutations of *TP53* [93].

Moreover, EBV-miR-BARTs are upregulated in EBV-positive HIV-related BL and could deregulate the host gene and miRNA expression in these tumors [17]. In particular, EBV-negative immunodeficiency-BL presents a downregulated expression of miR-BART6-3p compared with EBV-positive cases. This miRNA may have an impact on the proliferation, cell growth, and apoptosis in the BL cell line, downregulating the expression of *PTEN*, a negative regulator of the Akt/PI3K signaling pathway [94,95]. In addition, miR-BART6-3p regulates the expression of the IL-6 receptor (*IL-6R*), being able to contribute to immune system evasion [94]. Furthermore, it has been postulated that EBV-miRNAs have a role in the microenvironment regulation, reducing the innate and adaptative immune response in EBV-positive tumors, similar to BL (Figure 2) [96].

Moreover, EBERs induce the *IL-10* expression in EBV-BL cell lines, suggesting an EBV involvement in the tumor growth [97]. They bind protein kinase RNA-activated (PKR), a protein involved in interferon-α (IFN-α)-mediated apoptosis, avoiding its phosphorylation [98]. Therefore, EBERs can be also involved in apoptosis resistance.

#### 2.2.3. Impact of EBV on Clinical Features and Prognosis

To our knowledge, studies of the clinical features of EBV-positive BL specifically focused on PLWH have not been carried out. However, HIV-related BL has been more extensively studied. Patients usually develop BL early in HIV disease, and they still maintain a moderate amount of CD4+ lymphocyte counts (greater than 200 cells/µL), often without a history of opportunistic infections (Table 1) [81]. This lymphoma presents an aggressive clinical behavior in advanced stages (III and IV), with nodal and bone marrow involvement [29,99,100,101]. If relapse occurs, it is usually seen within the first year after response and can be associated with CNS involvement [102]. In the cART era, the prognosis of HIV-related BL has improved, and similar responses and survival probabilities are now achieved compared with those of non-HIV-infected individuals [103,104].

#### 2.2.4. Treatment

The treatment of HIV-related BL is based on intensive chemotherapeutic schemes as in the general population, but in combination with cART [84]. The chemotherapeutic strategies include cyclophosphamide, vincristine, doxorubicin, methotrexate/ifosfamide, etoposide, cytarabine (CODOX-M/IVAC) [105]; cyclophosphamide, methotrexate, dexamethasone, ifosfamide, vincristine, etoposide, cytarabine, doxorubicin, and vindesine in combination with rituximab (Burkimab) [103]; cyclophosphamide, vincristine, doxorubicin, and dexamethasone with methotrexate and high-dose cytarabine (hyper-CVAD); or dose-adjusted etoposide, prednisone, vincristine, cyclophosphamide, and doxorubicin with rituximab (DA-EPOCH-R) [106]. There is no standard second-line treatment for this lymphoma, and the few refractory/relapsed patients should be treated with experimental strategies (Table 2).

### 2.3. Plasmablastic Lymphoma

Plasmablastic lymphoma is a B-cell NHL that most commonly occurs in PLWH and is characterized by loss of GC B-cell markers and the expression of plasma cell markers and features of an activated B-cell (Figure 1) [29].

#### 2.3.1. Epidemiology

PBL is an uncommon lymphoma with an aggressive clinical course, with a median OS of 6–11 months [29,107]. This lymphoma presents more frequently in men (80%) with a median age of 39–46 years. It is closely related to HIV infection, representing around 2–12% of HRL [108,109,110,111,112] and in most cases the cells are also infected by EBV (75–100%) [113,114,115].

#### 2.3.2. Etiopathogenesis

The genetic and molecular characterization of PBL has not been clearly described. Nevertheless, recent studies have revealed that dysregulating mutations in the JAK-STAT and RAS-MAPK pathways are genetic signatures of PBL. These mutations mostly affect *STAT3*, *TP53*, and *RAS* family members, and with less frequency affect *MYC*, *EP300*, *CARD11*, *SOCS1*, and *TET2* [116,117,118,119]. Additionally, *MYC* translocation is the most frequent genetic alteration found [114,120,121], being more frequent in HIV-related PBL than in PBL in the general population (78% vs. 44%). In addition, it is more common in EBV-positive than in EBV-negative PBL (57–74 vs. 20–43%) [118,121,122]. The high frequency of EBV among HIV-related PBL reported in many studies indicates a strong association between the two viruses, and EBV latency I is the most common pattern (Table 1), but the latency III pattern has been observed as well [108,109,114,121,123,124]. Additionally, some studies describe different genetic alterations depending on the EBV status. Mutations in *TP53* are more frequent among EBV-negative compared with EBV-positive PBL [116,117], while mutations in *STAT3* and *SOCS1* are more common in EBV-positive cases [116]. Garcia-Reyero et al. reported that EBV-related PBL cases presented greater genomic stability than EBV-negative cases (87.5% vs. 54%), as well as a different mutational profile [118]. In this regard, *PRDM1* and *STAT3* mutations are only detected in EBV-related PBL, and most are also HIV-related [118]. Blimp1 is a transcriptional repressor of *MYC* in transformation of a B-cell to a plasma cell, so coexpression of both proteins would not be expected in this lymphoma [125]. However, both proteins are usually expressed in HIV-related PBL [122]. In this regard, Montes-Moreno et al. described that *PRDM1* mutations affect functional domains involved in the regulation of other genes, which could avoid the negative regulation of Myc, but not B-cell terminal differentiation. Thus, *PRDM1* mutations would reinforce Myc-driven lymphomagenesis [122]. This could also explain the discontinuation of plasmacytic differentiation at the plasmablast stage [126].

Given the biological similarities shared between PBL and BL extramedullary plasmacytoma (EMPC), a recent study revealed different EBV-miRNAs expression among these groups. A total of 38 EBV-miR-BARTs were upregulated in PBL compared with EMPC, while 19 EBV-miR-BARTs were downregulated in PBL compared with BL, suggesting that EBV-miRNAs could be useful for the differential diagnosis of PBL [127].

The expression of EBV lytic proteins in lymphomas could contribute to the lymphomagenesis. In this regard, PBL shows expression of EBV proteins related with lytic cycle such as BZLF-1/ZEBRA, BHRF-1/Ea-R, and BMRF-1/Ea-D, but absence of BLLF1/gp50 expression [127]. Other authors have recently described a higher expression of the lytic genes *BALF4* and *BALF5* in HIV-related PBL in EBV-positive cases [119].

EBV-related PBL shows an increased number of infiltrating T-cells, natural killer (NK) cells, and protumorigenic M2-macrophages, compared to EBV-negative PBL, and in turn presents higher activation of immune evasion mechanisms. In this regard, EBV-related PBL present an overexpression of programmed cell death ligand 1 (PD-L1) that is associated with immune evasion by tumoral cells [128]. These mechanisms could lead to the construction of a favorable microenvironment for lymphoma development.

#### 2.3.3. Impact of EBV on Clinical Features and Prognosis

Extranodal involvement is very frequent in PBL in PLWH (95% of cases) at diagnosis, and in these patients the lymphoma usually presents at advanced stages (III or IV) [108,109,112,115,120,129]. The oral cavity is the most frequent extranodal site (48–58%) [29,108,113,129]. Schommers et al. reported that some clinical features such as age older than 60, high serum lactate dehydrogenase, an Eastern Cooperative Oncology Group (ECOG) score > 2, and an International Prognostic Index (IPI) score of 2–5 are risk factors associated with worse outcomes [26]. The translocation of *MYC* has been associated with shorter OS compared to patients with non-rearranged *MYC* [114]. In one study, PLWL with *MYC* gene rearrangements were associated with a 6-fold increased risk of death [130]. Patients with EBV-related PBL have a worse prognosis than EBV-negative cases [112]. The elevated incidence of EBV infection could be useful for the differential diagnosis with other B-cell neoplasm with similar features.

While the prognosis of PBL has improved since the introduction of cART, these patients have a poor outcome, with a median OS of 10–15 months [107,108,129]. In this regard, the median CD4+ lymphocyte count at diagnosis is 63–165 cells/µL, indicating a status of important immunodeficiency (Table 1) [26,131,132]. Low CD4+ lymphocyte counts and the absence of cART treatment prior to lymphoma are considered factors of worse prognosis [112]. The impact of EBV on the prognosis of PBL is controversial, with some studies not reporting an influence of EBV infection on OS [26,108,124,129], whereas others have reported better outcomes in EBV-positive than in EBV-negative PBL in PLWH [114,124,133].

#### 2.3.4. Treatment

There is no standard treatment for PBL, although intensive treatment is recommended. PLWH patients are currently treated with the same regimens as those of the general population, in combination with cART, such as CODOX-M/IVAC, hyper-CVAD, or DA-EPOCH [112]. Plasmablastic differentiation of neoplastic cells has led to testing drugs against multiple myeloma. The proteasome inhibitor bortezomib has been used alone and in combination with other drugs. In this sense, the combination with DA-EPOCH has shown good results in a retrospective series, with a 5-year OS of 63% [134].

The role of ASCT in the treatment of PBL has not been well studied. It could be used as a first- or second-line treatment, although further studies are needed to confirm possible beneficial effects (Table 2) [135,136].

### 2.4. Primary Effusion Lymphoma

Primary effusion lymphoma is a rare and aggressive NHL appearing mainly in HIV-infected patients in the form of body cavity effusion, although there are also solid forms. The lymphoma cells have a plasma cell phenotype and are positive for HHV-8 [29], and elevated percentage of cases are also positive for EBV (Figure 1) [11,29,35,137,138]. The presence of EBV in the cells is a useful diagnostic tool to differentiate this lymphoma from HHV-8-positive DLBCL, not otherwise specified (NOS), a similar entity that overlaps histological features, since this entity is EBV-negative [29].

#### 2.4.1. Epidemiology

This lymphoma occurs mainly in men (90%) with a median age of 50–55 years and advanced AIDS disease. Two-thirds of the patients are HIV-infected (representing 3–5% of HIV-NHLs), and in this group the median age is 40–45 years, and around 70 years in HIV-negative individuals [29,138,139,140]. In addition, it is also closely related to EBV, which accounts for 70–100% of HIV-PEL [11,29,35,137,138].

This lymphoma also occurs in patients with other immunodeficiencies, such as liver cirrhosis and recipients of solid organ transplants. Moreover, some cases of PEL have been reported in elderly patients with positive serology for HHV-8 without any cause of immunodeficiency [141].

Approximately one-third of the patients have another HHV-8-related disease, such as Kaposi’s sarcoma, multicentric Castleman disease, or both [142].

#### 2.4.2. Etiopathogenesis

The neoplastic cells of PEL show low expression of mature B-cell genes and high expression of post-GC markers [29]. In addition, expression profile studies have revealed overexpression of plasma cell genes, indicating that the COO is a post-GC B-cell at an advanced stage of B-cell differentiation (Figure 1) [143].

HHV-8 is a DNA virus strongly implicated in the pathogenesis of PEL. This virus prevents apoptosis and activates NF-кB, promoting proliferation and indicating that HHV-8 is involved in PEL survival [144,145]. This virus has been related to the immune evasion system since it can increase the expression of PD-L1 [142]. In addition, most individuals with PEL are also coinfected by EBV. Latency I, with expression of EBNA1 and EBERs, is the most frequent subtype (90–92%), although it is possible to detect latency II (8%) with LMP1 expression in some cases (Table 1) [11,15,138,140]. The role of EBV in the pathogenesis of PEL remains unclear; however, the MAPK signaling pathway seems to differentiate EBV-positive and EBV-negative PEL, and together with HHV-8, could contribute to the proliferation and evasion of programmed cell death [10,57]. Curiously, Roy et al. have reported that EBV-negative PEL cell lines present more copy number variations than EBV-positive, thus indicating that EBV could maintain the genomic stability of host cells [146]. Nevertheless, the common expression of latency type I, without expression of immunogenic EBV proteins, suggests that EBV is not the only mechanism responsible for the pathogenesis of PEL [35,147,148]. In PEL cell lines, some EBV-miR-BARTs are expressed, but these cells do not express EBV-miR-BHRF1s. This miRNAs expression pattern is characteristic of EBV latency I [149]. It has been speculated that EBV cooperates with HHV-8 in the infection of B-cells, and HIV may contribute to lymphomagenesis, generating a permissive microenvironment [150].

In a mice model with both HHV-8 and EBV infection, HHV-8 increases the expression of EBV-lytic genes, especially *BZLF1* and *BALF2*, which lead to increase tumor growth. A high expression of EBV lytic genes has been also described in EBV and HHV-8 dual-infected patients with lymphoproliferative disorders, including PEL [151]. On the other hand, the EBV-protein EBNA-1 contributes to the survival of HHV-8 infected B-cells in PEL [152]. These results suggest a cooperation of both viruses (HHV-8 and EBV) in the lymphomagenesis of PEL.

#### 2.4.3. Clinical Features and Prognosis

Effusion(s) of body cavities (pleural, peritoneal, and/or pericardial) without tumor mass is the typical presentation of PEL [11,35]. By definition, all cases with this presentation (effusion) are at stage IV [139,140,153,154]. In addition, these patients usually present advanced AIDS disease with low CD4+ lymphocyte counts (median 98–133 cells/µL) (Table 1) [137,138,139,155]. Patients present with symptoms related to the affected cavity, with the pleura being the most frequent, followed by peritoneal and pericardial. Solid forms of PEL present with symptoms related to the affected organ, with the gastrointestinal tract being the most frequent [137,138].

The prognosis of PEL is poor, with a median OS of less than 12 months [29,137,154,156,157]. The prognostic factors of HIV-related PEL are controversial. Some authors report that the administration of cART before PEL development is a prognostic factor, since patients that received cART have a longer OS [137,158]. However, other authors did not observe this prolonged OS with cART administration [84]. In this regard, Boulanger et al. studied a series of 28 patients and reported that a bad performance status and absence of cART before lymphoma diagnosis were factors that had a negative influence on prognosis [137]. Furthermore, Lurain et al. described, in HIV-related PEL, an association between elevated interleukin-6 (IL-6) levels and shorter OS, while EBV infection correlated with longer survival [159].

#### 2.4.4. Treatment

There is no standard first-line treatment for PEL, and the available therapies present very poor results. Treatment with CHOP has achieved responses of 20–50% [155], while DA-EPOCH increases the complete response ratio and prolongs OS in patients with HIV-related PEL [140,159,160]. There is no established second-line treatment, but ASCT can be a therapeutic option, given the poor prognosis of this lymphoma [161,162]. Importantly, all of these treatments should be administered in combination with cART (Table 2).

### 2.5. Hodgkin Lymphoma

Hodgkin lymphoma is divided into two subtypes with different morphological and immunophenotypic features: nodular lymphocyte predominant HL and classic HL (cHL), which is related to PLWH [29,163,164,165]. In this lymphoma, Hodgkin Reed–Sternberg cells (HRS) are characteristically observed in a heterogeneous background of lymphocytes, eosinophils, neutrophils, macrophages, and plasma cells.

#### 2.5.1. Epidemiology

The incidence of HL in PLWH is around 50 per 100,000 cases/year, and has increased since the introduction of cART, with the risk being 5–25 times higher than in the general population [29,166]. The incidence of cHL has remained stable after the increase observed in the first years of the cART era [7,8,167]. At cHL diagnosis, CD4+ lymphocyte counts are moderately decreased (median between 150–260 cells/μL), indicating a non-severe immunosuppression (Table 1) [6,8,167,168,169,170,171]. Coinfection with EBV occurs in 90–100% of cases, compared to 30–40% in HIV-negative patients) [172]. In HIV-related cHL, mixed cellularity is the most common subtype, followed by lymphocyte depletion and nodular sclerosis [29,35,173].

#### 2.5.2. Etiopathogenesis

The malignant cells of cHL, which are HRS cells, seem to be GC-derived B-cells, as they carry somatic hypermutations, and this phenomenon is performed exclusively in the GC [174]. Therefore, aberrant mutations in BCR appear, and they are present in nearly all cHL. Unlike what occurs in normal conditions, these cells can escape from apoptosis. These events point to an origin in a preapoptotic GC-derived B-cell (Figure 1) [174,175].

Virtually all HIV-related cHLs are coinfected by EBV, and are latency II type, with expression of EBNA1, LMP1, and LMP2, as well as EBER and BART miRNAs. (Table 1) [176,177,178,179]. In this context, besides promoting cell cycle and inhibiting apoptosis, the LMP2 protein mimics BCR signaling and enables the survival of these cells in the absence of functional host BCR expression [179,180,181,182]. LMP1 promotes diverse signaling pathways involved in cell cycle progression, proliferation, and apoptosis, such as NF-кB, MAPK, PI3K/Akt, and JAK/STAT, and mimics CD40, a receptor necessary for B-cell activation [183,184]. In addition, EBV induces the overexpression of PD-L1 in HRS, leading to escape from immune response [185,186]. All of these mechanisms contribute to the proliferation and tumor progression of EBV-infected HRS. At the same time, HIV proteins such as gp120 and Tat reinforce chronic B-cell activation and promote the production of cytokines involved in the promotion of B-cell proliferation [187].

How the microenvironment influences the pathogenesis of cHL is not well understood. EBV-positive cHL frequently presents mixed cellularity with increased infiltration of NK cells, macrophages (M1 proinflammatory and M2 protumorigenic), CD4+ T-cells, and CTLs [188]. Although this tumor exhibits an increased infiltration of CTLs, EBV could inhibit their response by contributing to a permissive immunologic microenvironment [189]. Under immunodeficiency conditions, in cHL there is a depletion of CD4+ T-cells and an increased infiltration of M1 macrophages, and hence, HIV may also contribute to this permissive microenvironment [190]. Macrophage phenotypes could be regulated via EBV. Although the role of EBV-miRNAs has been scarcely studied so far in HL, EBV-BART miRNAs can be transferred to macrophages via exosomes, inducing the transformation of macrophages into a proinflammatory phenotype [191]. EBV-BART13-3p is the most expressed EBV-miRNA in HL, and this fact could transform macrophages and contribute to lymphoma microenvironment (Figure 2) [49].

#### 2.5.3. Impact of EBV on Clinical Features and Prognosis

Classical HL affecting PLWH is virtually always EBV-positive and is characterized by aggressive clinical characteristics, such as advanced stages (III/IV), bone marrow and multiple nodal involvement, and B symptoms. The proportion of males affected (80–98%) is higher than in HIV-negative subjects, and some studies have shown that the age at diagnosis is higher in PLWH, with a median age of 40–44 years, than in the general population [166,192,193,194].

The prognosis of HL in PLWH is similar to that of the general population using the same therapeutic strategies in both groups. The presence of EBV in the lymphoma cells has not been reported to have any impact on prognosis.

#### 2.5.4. Treatment

The treatment of cHL in PLWH is the same as that of the general population, based on chemotherapy with or without radiotherapy, in localized stages, and chemotherapy in advanced stages. The treatments most currently used are doxorubicin, bleomycin, vinblastine, and dacarbazine (ABVD) and bleomycin, etoposide, doxorubicin, cyclophosphamide, vincristine, procarbazine, and prednisone (BEACOPP) [195,196]. Relapses of cHL in PLWH can be treated with second-line drugs followed by ASCT (Table 2) [197,198].

## 3. Implications of EBV Load in HIV-Related Lymphoma

Several studies have evaluated the impact of EBV load on the diagnosis and prognosis of NHL and HL in the immunocompetent population [199,200]. In this regard, EBV loads measured in plasma and peripheral blood mononuclear cells (PBMCs) can serve as a diagnostic tool and could also have prognostic impact on lymphomas [201,202,203,204,205,206,207]. Fewer studies have been performed measuring EBV in peripheral blood of PLWH to determine its value as a diagnostic tool and prognostic factor. In this regard, the detection of EBV load has been reported to be more frequent among lymphomas in PLWH than in the general population [25,208,209]. Moreover, some studies, including ours, have suggested that EBV load measured in plasma could be a useful tool for the diagnosis of NHL and HL in PLWH, since EBV has been detected in plasma of these patients at lymphoma diagnosis [210,211]. This fact has also been demonstrated in PBMCs and serum in HRL [212]. In our recent study by Muncunill et al., we reported a strong association between the detection of EBV load in plasma and HIV-related lymphoma, since EBV was significantly less frequently detected in both HIV-negative patients with lymphoma and HIV-infected individuals without lymphoma [25,213]. This association has also been reported in whole blood (WB) [214]. Additionally, EBV loads measured either in plasma [25,210,211,214] or in PBMCs [215] could be useful to anticipate the development of lymphoma in PLWH. In contrast, EBV load measured in PBMCs was not useful for predicting the development of HIV-related NHL (DLBCL included) in the study of Van Baarle et al. [216]. Different studies suggest that EBV load measured in plasma [25,211,213,217], WB [217], PBMCs, and serum [212] can be used as a follow-up biomarker in HRL. All these techniques can be used to detect the presence of EBV in peripheral blood and have demonstrated to be useful for the follow-up of lymphomas in PLWH.

Some studies have pointed out that the presence of EBV in peripheral blood could be used as a prognostic factor in HRL. Individuals with high levels of EBV load in plasma have shown worse OS [25,209,210,211]. In this regard, Muncunill et al. reported a negative impact of high EBV load on the survival of HIV-related NHL treated without rituximab. However, this difference in outcome was not observed in patients treated with rituximab [25]. On the other hand, the impact of plasma EBV load in HL of PLWH remains controversial. In an HIV-cohort published by Muncunill et al., high EBV load in plasma had a negative influence on the prognosis of HL patients [25]. In contrast, in other cohorts the EBV plasma load was not described as having any prognostic impact on the survival of HIV-related HL [217,218,219].

In summary, the results of the different studies point out that the EBV load in peripheral blood may be a diagnostic tool and a biomarker for HRL. Further studies with larger cohorts of NHL and HL in PLWH are needed to clarify the usefulness of EBV in peripheral blood as a prognostic factor in these patients.

## 4. General Recommendations for the Treatment of Lymphomas in PLWH

Several studies have shown similar responses and prognoses in all lymphoma subtypes, regardless of HIV infection, when patients are treated with standard therapies. Therefore, the same chemotherapeutic strategies used in the general population are currently recommended for PLWH [168]. As previously stated in the different sections of the article, EBV-positive lymphomas in PLWH are currently treated following the general recommendations for lymphomas in these patients. Moreover, the same additional measures and supportive care recommended for treatment of lymphomas in PLWH should be applied to EBV-positive cases.

Most authors recommend concomitant administration of cART during lymphoma treatment due to the evidence that better outcomes are obtained with this approach [220]. Nevertheless, the synergistic toxicity and the drug–drug interactions between multiagent chemotherapy and cART, especially protease inhibitors, should be considered before starting lymphoma treatment [221,222,223]. Thus, interdisciplinary collaboration between hemato-oncologists and HIV specialists is the best way for the optimal treatment of both lymphoma and HIV infection while minimizing the risk of adverse outcomes for the patients.

Although there is a lack of studies on the efficacy of CNS prophylaxis in lymphomas affecting PLWH, patients with lymphomas at high risk of CNS involvement should receive prophylaxis as recommended for the general population. Therefore, patients with BL, *MYC*-rearranged high-grade lymphomas, PBL, as well as DLBCL with risk factors for meningeal involvement, should receive additional intrathecal prophylaxis or high-dose methotrexate, depending on the patient tolerability and the experience of the team [224,225,226,227].

Primary infectious prophylaxis using colony-stimulating factors, such as G-CSF given after every cycle of chemotherapy, is highly recommended to prevent neutropenia and dose reductions [73,228,229]. Moreover, common infections affecting PLWH should be prevented because chemotherapy decreases the CD4+ lymphocyte. In this regard, prophylaxis against *Pneumocystis jirovecci* [222,230,231] is recommended, and *Mycobacterium avium* complex should also be prevented in patients with CD4+ lymphocytes lower than 50/µL [73,229,232]. Moreover, hepatitis B and C infections are common among HIV-infected individuals. In these cases, concomitant treatment with antiviral therapy against HBV and HCV must be considered [73,223].

## 5. EBV-Targeted Therapies

EBV status is currently not a differential factor for the choice of treatment in HRL, and patients are treated with the same therapeutic strategies independently of the presence of EBV in lymphoma. Nevertheless, given that EBV is involved in the pathogenesis of HRL, different strategies targeting EBV could improve the treatment of these patients. In this regard, preclinical studies have evaluated different strategies in lymphoma cell lines based on protein inhibitors of signaling pathways deregulated by EBV, such as BCR signaling, PI3K, JAK/STAT, MAPK, NF-кB, cell cycle, and apoptosis [28]. Other studies have focused on the design of drugs against EBV antigens, such as EBNA1 [233,234]. Moreover, some strategies are based on immunotherapy, including PD-1/PD-L1 antibodies, monoclonal antibodies, or T-cell receptor-modified T-cell therapies, among others [235,236,237,238,239,240]. Lastly, several studies attempting to develop a vaccine against EBV have been performed. However, there is still no commercial vaccine against this virus [241].

Despite the extensive in vitro studies of EBV targets, we still have a long wait until the development and approval of EBV-specific therapies in EBV-related lymphomas. Only a few clinical trials on EBV-related lymphomas have been performed so far (Table 2). Some have evaluated drugs based on the combination of nucleoside analogs that inhibits viral DNA polymerase in the lytic phase of viral replication, such as ganciclovir (GCV) and zidovudine (azidotimidine) in combination with immunomodulatory IL-2 and cART in EBV-positive HIV-related PCNSL [242,243]. The results revealed better OS and could be effective for the treatment of these patients. Specifically, GCV reduced the EBV-DNA load in CSF of patients with HIV-PCNSL, improving survival [244]. Unfortunately, latent EBV tumors do not express the EBV-thymidine kinase (TK), and for this reason, GCV may be ineffective. Thus, other strategies are focused on the induction of the EBV lytic cycle and EBV-TK [245]. In this regard, clinical trials using arginine butyrate in combination with GCV in refractory EBV-related lymphomas have shown good tolerability and antitumoral response (10 of 15 patients; 4 complete and 6 partial responses), although these results should be confirmed in a larger cohort [246,247]. On the other hand, the inhibition of histone deacetylases (HDACs) also may induce lytic infection from the latency state. Some HDAC inhibitors such as vorinostat/suberoylanilide hydroxamic acid (SAHA) have been tested in HL and NHL [248,249]. These studies showed that SAHA could have a positive effect on HL and DLBCL, leading to partial response or stable disease, as well as a modest effect on relapsed DLBCL, although these results require validation. Currently, an ongoing phase I/II clinical trial is testing the combination of HDAC inhibitors and valganciclovir in relapsed/refractory EBV-related lymphomas.

Regarding immune evasion, PD-L1 is overexpressed in diverse EBV-related lymphomas, and therefore, strategies targeting PD-1/PD-L1 interaction are of great importance and could be a very useful target for the treatment of EBV-related HRL. A clinical trial of immunocompetent HL patients revealed that the blockage of PD-1, using the monoclonal antibody pembrolizumab, could be useful for the treatment of HL [250]. Currently, several clinical trials are evaluating the effect of PD-1/PD-L1 inhibitors, such as nivolumab, pembrolizumab, toripalimab, and sintilimab, on EBV-related NHL.

Lastly, EBV-specific T-cells (EBVST) that recognize specific EBV antigens presented by infected B-cells are another EBV-targeted therapeutic strategy. LMP1/LMP2- or LMP2-specific CTLs administered to 50 patients with EBV-related NHL or HL showed promising results in patients with risk or refractory/relapsed disease, with most achieving a 2-year event-free survival. On the other hand, 62% of patients with active lymphoma achieved complete or partial response with EBVST administration [251]. EBNA1-specific CTL administration in patients with post-transplant proliferative disease after stem cell transplantation seems to restore T-cell immune response against EBV [252]. Transfusion of EBVST may restore immune response in EBV-related lymphoma patients and results in a promising therapy to eliminate EBV-infected B-cells and avoid possible relapses in EBV-positive HRL.

## 6. Conclusions

In summary, EBV is involved in the lymphomagenesis of the different HRL subtypes mediated by several viral molecules. Close cooperation between EBV and HIV, as well as HHV-8 in some lymphoma subtypes, seems to be an additional lymphomagenic mechanism in which HIV may favor a permissive microenvironment for EBV infection and the development of lymphoma. EBV load in peripheral blood can be used as a lymphoma biomarker in PLWH. Diverse EBV-targeted therapies have reported promising results for the treatment of EBV-related lymphomas. Nevertheless, most studies did not include PLWH, and therefore further clinical trials are needed to confirm these results in HRL. Given the postulated cooperation between EBV and HIV in the etiopathogenesis of these lymphomas, the combination of new therapeutic strategies against both viruses should be considered in order to improve the adverse outcomes that a great proportion of these patients still have.

## Figures and Tables

**Table 1:** Lymphomas affecting PLWH. The percentage of EBV, latency type, and the CD4+ lymphocyte counts for each lymphoma are shown. BL: Burkitt lymphoma; PBL: plasmablastic lymphoma; DLBCL: diffuse large B-cell lymphoma; EBV: Epstein–Barr virus; HL: Hodgkin lymphoma; PCNSL: primary central nervous system lymphoma; PEL: primary effusion lymphoma.

Lymphoma	EBV Frequency	EBV Latency Type	CD4+ Lymphocyte Counts (Cells/µL)
DLBCL	30–50%	I/II/III	100–223
PCNSL	80–100%	III	<50
BL	30–60%	I	200–270
PBL	75–100%	I	63–165
PEL	70–100%	I	98–133
HL	90–100%	II	150–200

**Table 2:** Current therapeutic regimens of HRL and EBV-targeted therapies. (**A**). Chemotherapeutic regimens of 1st and 2nd line in combination with cART for the treatment of HRL associated with EBV infection. (**B**). Diverse EBV-targeted therapeutic strategies in preclinical studies (left) and EBV-targeted therapies tested in clinical trials in EBV-related lymphomas (right). ABVD: doxorubicin, bleomycin, vinblastine, and dacarbazine; ASCT: autologous stem cell transplantation; AZT: azidotimidine; BEACOPP: bleomycin, etoposide, doxorubicin, cyclophosphamide, vincristine, procarbazine, prednisone; BL: Burkitt lymphoma; Burkimab: cyclophosphamide, methotrexate, dexamethasone, ifosfamide, vincristine, etoposide, cytarabine, doxorubicin, vindesine; cART: combined antiretroviral therapy; CAR-T: chimeric antigen receptor T-cells; CHOP: cyclophosphamide, doxorubicin, vincristine, and prednisone; CODOX-M/IVAC: cyclophosphamide, doxorubicin, vincristine, methotrexate/ifosfamide, etoposide, cytarabine; CTLs: cytotoxic T-lymphocytes; DA-EPOCH: dose-adjusted etoposide, prednisone, vincristine, cyclophosphamide, and doxorubicin; DHAP: dexamethasone, cytarabine, and cisplatin; DHAX: dexamethasone, cytarabine, and oxaliplatin; DLBCL: diffuse large B-cell lymphoma; EBNA1: EBV-nuclear antigen 1; EBV: Epstein–Barr virus; EBVST: EBV-specific T-cells; ESHAP: etoposide, cisplatin, methylprednisolone, and cytarabine; GCV: ganciclovir; GDP: gemcitabine, dexamethasone, and oxaliplatin; GEMOX: gemcitabine and oxaliplatin; HDAC: histone deacetylase; HD-MTX: high dose methotrexate; HIV: human immunodeficiency virus; HL: Hodgkin lymphoma; Hyper-CVAD: cyclophosphamide, doxorubicin, vincristine, dexamethasone with methotrexate and high-dose cytarabine; ICE: ifosfamide, etoposide, and carboplatin; IC-HL: immunocompetent Hodgkin lymphoma; LMP1: latent membrane protein-1; LMP2: latent membrane protein-2; NHL: non-Hodgkin lymphoma; PBL: plasmablastic lymphoma; PCNSL: primary central nervous system lymphoma; PD-1: programmed death 1; PD-L1: programmed death ligand 1; PEL: primary effusion lymphoma; PTLD: post-transplant lymphoproliferative disorder; R: rituximab; SAHA: suberoylanilide hydroxamic acid; SCT: stem cell transplantation; THP-COP: pirarubicin, cyclophosphamide, vincristine, and prednisolone.

(A) Chemotherapeutic Treatment in HIV-Related Lymphomas		
**Lymphoma**	**1st Line**	**2nd Line**
DLBCL	R-CHOP	R-ESHAP, R-ICE, R-GEMOX; followed by ASCT
PCNSL	HD-MTX	ASCT, Radiotherapy
BL	CODOX-M/IVAC Burkimab Hyper-CVAD DA-EPOCH-R	R-DHAP, R-DHAX, R-GDP, R-GDP, R-GEMOX
PBL	CODOX- M/IVAC Hyper-CVAD DA-EPOCH-R Bortezomib	THP-COP, ESHAP, ICE and ASCT
PEL	DA-EPOCH CHOP	ASCT, radiotherapy, bortezomib
HL	ABVD BEACOPP	ESHAP, DHAP, ICE; followed by ASCT
**(B) EBV-Targeted Therapies**		
**Preclinical Studies**		
Small molecule inhibitors	• Targeting host factors and signaling pathways • Targeting EBV antigens	
Immunotherapy	• PD-1/PD-L1 antibodies • Monoclonal antibodies • EBVST • T-cell receptor-modified T-cell therapy • CAR-T	
**Clinical Trials**		
Antiretroviral drugs	• GCV and AZT in combination with immunomodulatory IL-2 and cART in EBV+ HIV-related PCNSL	
Induction of lytic infection from latency state	• Arginine butyrate in combination with GCV in refractory EBV-related lymphomas • Inhibition of histone deacetylase (HDAC), such as SAHA in HL and NHL	
Inhibition of PD-1/PD-L1	• Prembrolizumab in IC-HL	
EBVST	• LMP1/LMP2- or LMP2-specific CTLs administered in EBV-related NHL or HL • EBNA-specific CTL in patients with EBV-related PTLD after SCT	
